# Intraventricular central neurocytoma molecularly defined as extraventricular neurocytoma: a case representing the discrepancy between clinicopathological and molecular classifications

**DOI:** 10.1007/s10014-023-00469-2

**Published:** 2023-09-11

**Authors:** Daisuke Sato, Hirokazu Takami, Shunsaku Takayanagi, Masako Ikemura, Reiko Matsuura, Shota Tanaka, Nobuhito Saito

**Affiliations:** 1 Department of Neurosurgery, The University of Tokyo Hospital, 7-3-1 Hongo, Bunkyo-Ku, Tokyo, 113-8655 Japan; 2 Department of Pathology and Diagnostic Pathology, Graduate School of Medicine, The University of Tokyo, Tokyo, Japan

**Keywords:** Central neurocytoma, Extraventricular neurocytoma, DNA methylation profile, FGFR1-TACC-1, Molecular classification

## Abstract

Central neurocytoma (CN) is classically defined by its intraventricular location, neuronal/neurocytic differentiation, and histological resemblance to oligodendroglioma. Extraventricular neurocytoma (EVN) shares similar histological features with CN, while it distributes any site without contact with the ventricular system. CN and EVN have distinct methylation landscapes, and EVN has a signature fusion gene, *FGFR1*-*TACC1*. These characteristics distinguish between CN and EVN. A 30-year-old female underwent craniotomy and resection of a left intraventricular tumor at our institution. The histopathology demonstrated the classical findings of CN. Adjuvant irradiation with 60 Gy followed. No recurrence has been recorded for 25 years postoperatively. RNA sequencing revealed *FGFR1*-*TACC1* fusion and methylation profile was discrepant with CN but compatible with EVN. We experienced a case of anatomically and histologically proven CN in the lateral ventricle. However, the FGFR1-TACC1 fusion gene and methylation profiling suggested the molecular diagnosis of EVN. The representative case was an “intraventricular” neurocytoma displaying molecular features of an “extraventricular” neurocytoma. Clinicopathological and molecular definitions have collided in our case and raised questions about the current definition of CN and EVN.

## Introduction

Central neurocytoma (CN) is a rare neoplasm predominantly arising from the supratentorial ventricular system [3]. The “honeycomb pattern” composed of uniform tumor cells with small clear round features, neuropil islands, calcifications, and neuronal/neurocytic differentiation are the hallmarks of CN [6]. The partial histological resemblance to oligodendroglioma has been noted since its first description [6], while the neuronal/neurocytic differentiation observed in CN is distinct from oligodendroglioma. Although several chromosomal and genetic aberrations have been observed, the distinguishing molecular features of CN have not been well-characterized for decades [1]. Recently, methylation profiling has emerged as a powerful tool in confirming the diagnosis of CN and enabled differentiation from other tumors with similar histological features [8]. Extraventricular neurocytoma (EVN) is a clinicopathological entity that presents similar histopathological features to CN, albeit it arises from extra-ventricular parenchymal tissue [19]. According to the 5th edition of WHO classification, it is defined as “arise in almost any location in the CNS without contact with the ventricular system” in accordance with its nomenclature. The copy number profile and DNA methylation landscape of EVN differ from CN, and the *FGFR1*-*TACC1* fusion is a striking, distinctive feature of EVN [19]. We experienced a case of anatomically and histologically proven CN present in the anterior part of the left lateral ventricle. However, *FGFR1*-*TACC1* fusion was detected by RNA sequence, and methylation profiling indicated the diagnosis of EVN. Thus, the representative case was an “intraventricular” neurocytoma harboring molecular features of “extraventricular” neurocytoma. Namely, clinicopathological and molecular definitions have collided in our case.

## Clinical summary

**Fig. 1:**
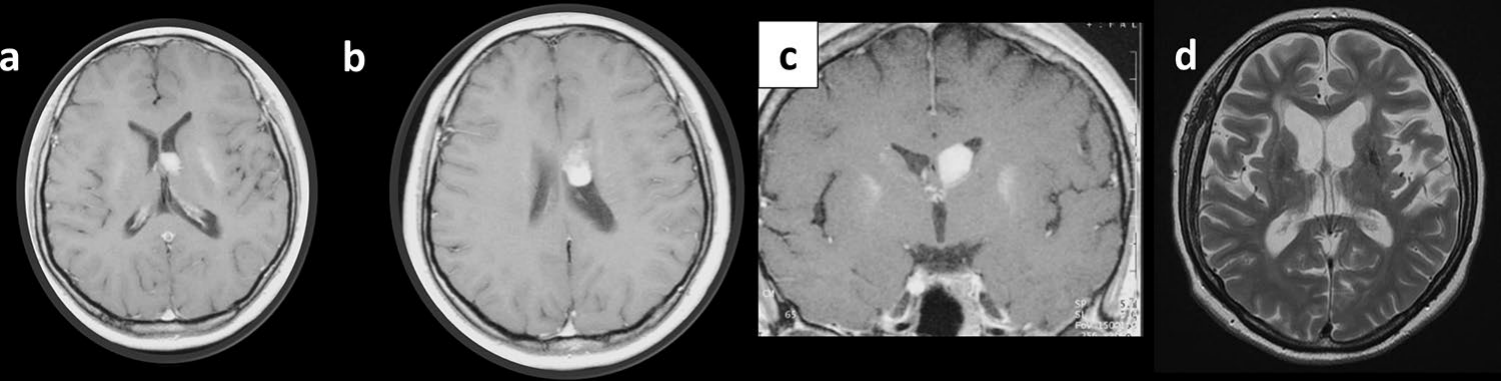
Magnetic resonance imaging (MRI) revealed a solid mass with strong contrast enhancement located at the left lateral ventricle (**a**). The lesion was attached to the corpus callosum and the caudate nucleus (**b**). The lesion occupied the anterior horn of the left lateral ventricle (**c**). Follow-up imaging revealed no tumor recurrence 25 years post-operatively (**d**)

A 30-year-old female presented with a mild headache lasting for a month. She was neurologically intact on physical examination. Magnetic resonance imaging (MRI) revealed a mass of 22 mm in size occupying the anterior horn of the left lateral ventricle (Fig. 1a–c). The lesion was attached to the caudate head and septum pellucidum. Hydrocephalus was not evident. For diagnostic as well as mass reduction purposes, tumor resection was performed. After frontoparietal craniotomy and interhemispheric approach, callosotomy led to the opening of the lateral ventricle. Under a microscope, the tumor partly invaded the head of the caudate nucleus and the corpus callosum and was moderately adhered to the septum pellucidum. Gross total resection was achieved. The postoperative course was uneventful.

After reaching the histological diagnosis of central neurocytoma, irradiation with 60 Gy for the tumor-originating site was completed. Regular follow-up with imaging revealed no postoperative tumor recurrence for 25 years (Fig. 1d). The patient provided written informed consent for the case to be reported.

## Pathological findings

**Fig. 2:**
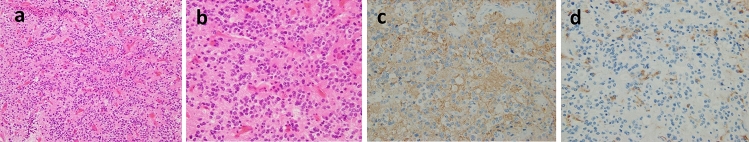
Fairly uniform round cells were interspersed by fine branching vasculature, which formed perivascular pseudorosettes (**a**). Tumor cells had oval nuclei with fine chromatin and occasional nucleolus. Neuropil-like matrix was observed in the background (**b**). Tumor cells were positive for synaptophysin (**c**), and negative for GFAP (**d**)

Histopathological evaluation revealed sheets of fairly uniform round cells interspersed by fine branching vasculature. Tumor cells were partially arranged radially around blood vessels, which formed perivascular pseudorosettes (Fig. 2a). Tumor cells exhibited oval nuclei with stippled chromatin and occasional nucleolus. Neuropil-like matrix was observed in the background (Fig. 2b). Immunohistochemical staining was positive for synaptophysin (Fig. 2c), and negative for GFAP (Fig. 2d). Taken together, a histological diagnosis of central neurocytoma was made.

**Fig. 3:**
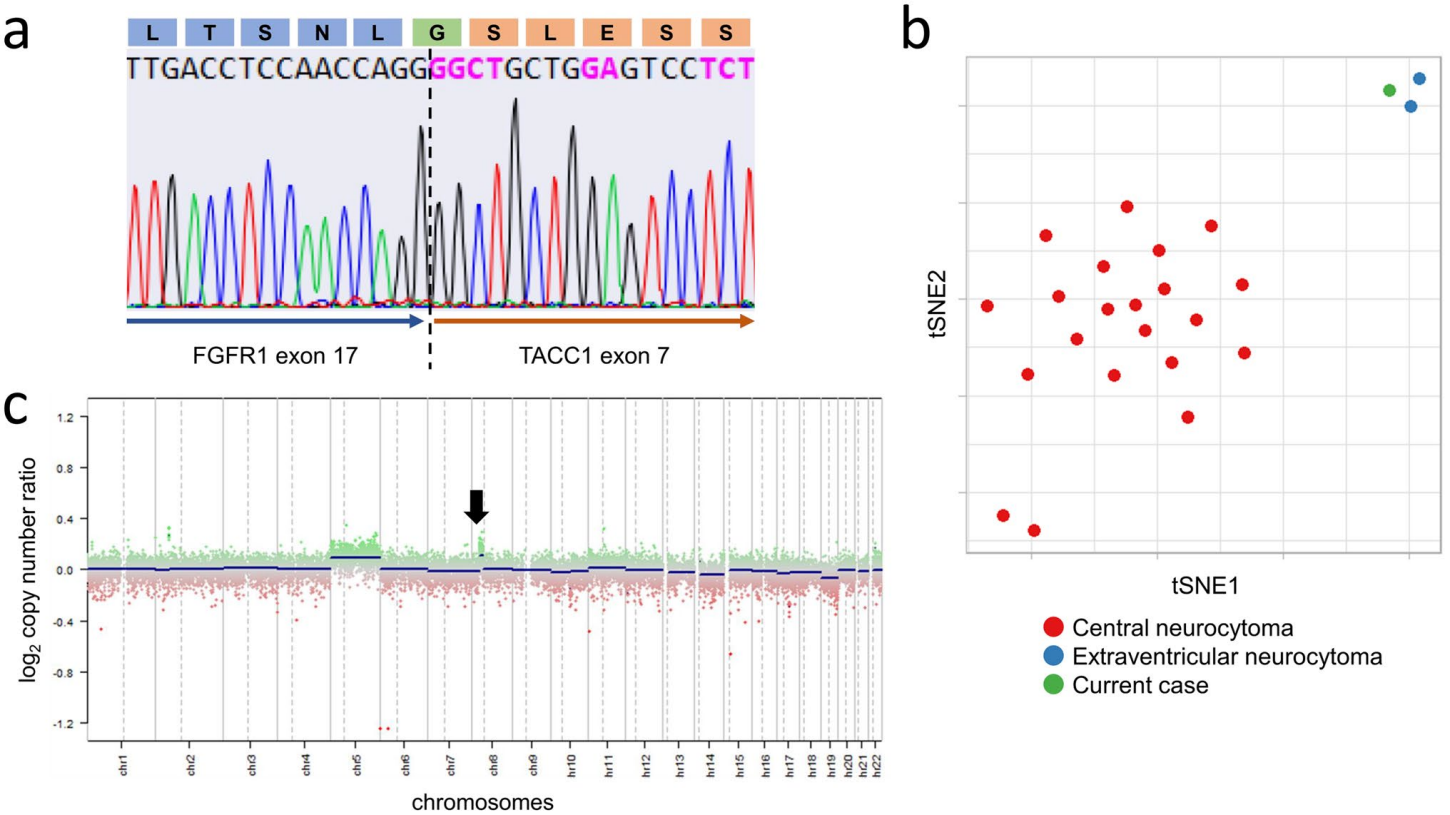
Sanger sequencing revealed the in-frame fusion of FGFR1 exon 17 and TACC1 exon 7 (**a**). tSNE analysis showed that the current case was clustered with two extraventricular neurocytoma cases and obviously separated away from 21 central neurocytoma cases (**b**). Copy number analysis demonstrated an overall flat profile, except gain in the entire chromosome 5 and partial gain in the short arm of chromosome 8 (**c**). The gain in chromosome 8 corresponds to the *FGFR1*-*TACC1* fusion (arrow)

According to the manufacturer's protocols, DNA and RNA were extracted from the frozen tumor sample using QIAamp DNA mini kit (Qiagen, Tokyo, Japan) and miRNeasy Mini Kit (Qiagen). First-strand cDNA was synthesized from 500 ng total RNA with Superscript IV (Invitrogen Life Technologies, Carlsbad, CA). RNA sequencing and data analysis were performed as described previously [21]. *FGFR1*-*TACC1* fusion was discovered, which was validated on Sanger sequencing using the following primer pair set on cDNA: forward primer 5′-CTGTACATGATGATGCGGG-3′ and reverse primer 5′-TTCTCTTATTAAGGTGAGCACG-3′ (Fig. 3a). DNA was analyzed using the Illumina Infinium Human Methylation EPIC Bead Chip array according to the manufacturer’s protocol. The methylation profiling classifier developed by the German Cancer Research Center (DKFZ)/University Hospital Heidelberg/German Consortium for Translational Cancer Research (DKTK) (DKFZ classifier, molecularneuropathology.org) was applied via their website (Brain tumor classifier; Version: 12.5) [21]. It categorized this case as the methylation class of “extraventricular neurocytoma,” with a calibrated score of 0.99. IDAT files for 21 CN cases and 2 EVN cases were downloaded from published studies (GSE 90496, 152,653) and integrated into our analysis. Raw signal intensities were obtained from IDAT data files and processed with minfi Bioconductor package version 1.44.0 (R v4.2.2) to calculate β values as described previously [14]. For tSNE, default parameters were used, except for perplexity = 8 (Rtsne v0.16) [21]. The top 5000 probes of standard deviation (SD) were used, and tSNE showed that our case was clustered with the other two EVNs away from CN cases (Fig. 3b). Copy number profiles were determined using the Conumee package (v1.9.0) [17]. Copy number was overall flat, except for gains in entire chromosome 5 and part of chromosome 8p (Fig. 3c). Ultimately, a molecular diagnosis of EVN was made. Taken in concert the intraventricular occurrence of the tumor, the integrative diagnosis was extraventricular neurocytoma, not elsewhere classified.

## Discussion

A case of anatomically and histologically diagnosed intraventricular CN with molecular features of EVN was presented. Though the characteristic histological findings such as monomorphic, regular nuclear morphology, and neuronal/neurocytic differentiation were consistent with the diagnosis of CN, the presence of distinct fusion revealed by RNA sequencing and methylation profiling clearly corroborated the diagnosis of EVN. The tumor was located in the left lateral ventricle; it was an “intraventricular” neurocytoma and was in an apparent contradiction to the nomenclature “extraventricular” neurocytoma.

The current case showed radiographical characteristics concordant with the classic presentation of CN. CN is postulated to originate from bipotential progenitor cells embedded in the periventricular matrix [22]. Therefore, relatively small tumors show a broad base attached to the lateral ventricular wall, and only partially adheres to the septum pellucidum [5, 16, 18]. Meanwhile, in medium-sized to large tumors, the septum pellucidum is often involved, and significant adherence is frequently observed. Hence, as the CN increase in size, the tumor originating from the periventricular matrix grows toward the septum pellucidum and the contralateral lateral ventricle [5, 16, 18]. Our case demonstrated a broad base related to the lateral ventricle wall, with only mild adherence to the septum pellucidum. Moderate contrast enhancement was also observed. These features were consistent with the characteristics of small-sized CN. In contrast, EVN is a tumor that can arise from any location within the parenchyma and is exclusively located outside the ventricle system [19]. EVN demonstrates a variable appearance compared to that of CN, although they are largely similar [7]. The lesion in our case was mainly located intraventricularly, with only a subtle extra-ventricular portion, which could be interpreted as the origin site of the CN. Therefore, our case exhibited strong concordance with the well-known radiographical features of CN. However, the molecular diagnosis showed a definite discrepancy with the clinical and radiological diagnosis.

We postulate two hypotheses to explain this phenomenon: one is the possibility that an EVN emerged close to the ventricular system and mainly grew into it instead of invading the brain parenchyma. EVN may arise at any site in the intracranial compartment [19]. Another hypothesis is that CN can present with molecular features of EVN. Next-generation sequencing and methylation profiling modified the histological diagnosis of CN into ganglioglioma in two cases in a certain cohort [8]. In addition, methylation profiling modified the histological diagnosis of EVN into diffuse leptomeningeal glioneuronal tumor, rosette-forming glioneuronal tumor, pilocytic astrocytoma, oligodendroglioma, astrocytoma, diffuse midline glioma H3K27M-mutant and glioblastoma in thirteen cases in another cohort [19]. Although much less likely, taken in concert with the trend toward more emphasis on molecularly based diagnosis, we presume that definitive histological diagnosis of CN and EVN still may include heterogeneous molecular pathologies.

In fact, the treatment course would not have been different whether the diagnosis was CN or EVT in the current case. A maximal safe resection is the cornerstone of the treatment for both tumors [15], and gross-total resection provides an excellent prognosis and minimizes the chances of recurrence [11]. The overall survival of CN is 95.3% (mean follow-up 90.5 months) for gross-total resection [12], and the 5-year overall survival rate of EVN without atypia is reported as 96% [9]. In cases with subtotal resection, postoperative adjuvant radiotherapy can prevent tumor progression and recurrence in both tumors. We treated the patient with maximal safe resection and postoperative adjuvant radiotherapy without tumor recurrence for 25 years.

However, most histologically proven EVN patients possess the fusion gene *FGFR1*-*TACC1* [19]. *FGFR* has garnered attention as a therapeutic target recently [4]. Irreversible inhibitor of FGFR has developed for the treatment of several solid cancers; cholangiocarcinoma, breast cancer, gastric cancer, urothelial cancer, oesophageal cancer, and non-small cell lung cancer, especially for previously treated, unresectable cases [13, 20]. Hence, EVN has the potential to be treated with novel monoclonal antibodies. We, therefore, pose that distinguishing the biological behavior of CN and EVN not by the histopathological features but by the molecular markers would provide therapeutic benefits. Importantly, *FGFR* alteration is not specific for EVN but is also observed in diffuse astrocytoma, pilocytic astrocytoma, dysembryoplastic neuroepithelial tumor, pleomorphic xanthoastrocytoma, polymorphous low-grade neuroepithelial tumor of the young, and glioblastoma [2, 10]. Integrative diagnosis based on imaging, pathological and molecular findings is thus important.

Molecularly diagnosed “extraventricular” neurocytoma in the ventricular system was presented. Imaging, pathological diagnosis, and molecular diagnosis may show a mismatch. Our case raises questions about the current definition of CN and EVN, and further reclassification of neurocytoma may provide therapeutic benefits for certain patients.

## Data Availability

The data sets generated during the current study are available from the corresponding author upon reasonable definitive request.

## Abbreviations

CN: Central neurocytoma
CT: Computed tomography
EVN: Extraventricular neurocytoma
WHO: World Health Organization
